# *CRK12*: A Key Player in Regulating the *Phaseolus vulgaris*-*Rhizobium tropici* Symbiotic Interaction

**DOI:** 10.3390/ijms241411720

**Published:** 2023-07-21

**Authors:** Antonino M. Lecona, Kalpana Nanjareddy, Lourdes Blanco, Valeria Piazza, José Antonio Vera-Núñez, Miguel Lara, Manoj-Kumar Arthikala

**Affiliations:** 1 Ciencias Agrogenómicas, Escuela Nacional de Estudios Superiores Unidad León, Universidad Nacional Autónoma de México (UNAM), León 37689, GTO, Mexico; antonino.lecona@comunidad.unam.mx (A.M.L.); kalpana@enes.unam.mx (K.N.); 2 Departamento de Biología Molecular de Plantas, Instituto de Biotecnología, Universidad Nacional Autónoma de México (UNAM), Cuernavaca 62210, MOR, Mexico; lourdes.blanco@ibt.unam.mx (L.B.); miguel.lara@ibt.unam.mx (M.L.); 3 Centro de Investigaciones en Óptica A. C., Loma del Bosque 115, León 37150, GTO, Mexico; 4 Departamento Biotecnología, Centro de Investigación y de Estudios Avanzados, Unidad Irapuato, Irapuato 36821, GTO, Mexico; jose.vera@cinvestav.mx

**Keywords:** cysteine-rich receptor-like kinases, CRK, hyper nodulation, nitrogen fixation, overexpression, *Phaseolus*, senescence, silencing, *Rhizobium*, Symbiosis

## Abstract

Cysteine-rich receptor-like kinases (CRKs) are a type of receptor-like kinases (RLKs) that are important for pathogen resistance, extracellular reactive oxygen species (ROS) signaling, and programmed cell death in plants. In a previous study, we identified 46 CRK family members in the *Phaseolus vulgaris* genome and found that *CRK12* was highly upregulated under root nodule symbiotic conditions. To better understand the role of *CRK12* in the *Phaseolus*–*Rhizobia* symbiotic interaction, we functionally characterized this gene by overexpressing (*CRK12*-OE) and silencing (*CRK12*-RNAi) it in a *P. vulgaris* hairy root system. We found that the constitutive expression of *CRK12* led to an increase in root hair length and the expression of root hair regulatory genes, while silencing the gene had the opposite effect. During symbiosis, *CRK12*-RNAi resulted in a significant reduction in nodule numbers, while *CRK12*-OE roots showed a dramatic increase in rhizobial infection threads and the number of nodules. Nodule cross sections revealed that silenced nodules had very few infected cells, while *CRK12*-OE nodules had enlarged infected cells, whose numbers had increased compared to controls. As expected, *CRK12*-RNAi negatively affected nitrogen fixation, while *CRK12*-OE nodules fixed 1.5 times more nitrogen than controls. Expression levels of genes involved in symbiosis and ROS signaling, as well as nitrogen export genes, supported the nodule phenotypes. Moreover, nodule senescence was prolonged in *CRK12*-overexpressing roots. Subcellular localization assays showed that the *Pv*CRK12 protein localized to the plasma membrane, and the spatiotemporal expression patterns of the *CRK12*-promoter::GUS-GFP analysis revealed a symbiosis-specific expression of *CRK12* during the early stages of rhizobial infection and in the development of nodules. Our findings suggest that CRK12, a membrane RLK, is a novel regulator of *Phaseolus vulgaris-Rhizobium tropici* symbiosis.

## 1. Introduction

Receptor-like kinases (RLKs) are abundant proteins located in the plasma membrane of various eukaryotic organisms, as evidenced by several studies [1,2,3]. Within the RLK family, cysteine-rich receptor-like kinases (CRKs) or domains of unknown function (DUF26) proteins represent a significant subgroup. CRKs consist of distinct domains, including an extracellular domain responsible for perceiving signals, a transmembrane domain, and an intracellular serine/threonine (Ser/Thr) protein kinase domain that facilitates signal transduction [4]. The classification of CRK proteins is based on domain types, such as DUF26 and Ginkbilobin-2 (Gnk2), or the presence of antifungal domain motifs in the extracellular region [5].

DUF26 domains possess a unique structure comprising of two α-helices and a five-stranded β-sheet, along with three conserved cysteine residues arranged in a C-X(8)-C-X(2)-C configuration, capable of forming two cysteine bridges. The cysteine bridges in DUF26 domains allow CRKs to sense reactive oxygen species (ROS)/redox signals (similar to other cysteine-rich domains) before transmitting the signal through the cytoplasmic kinase domain [6]. Plant CRKs exhibit considerable diversity, with examples such as 30 members in *Gossypium* [7], over 40 members in *Oryza* [8], 44 in *Arabidopsis* [4,9], 46 in *Phaseolus* [10], and 91 in *Glycine* [11].

CRKs have been implicated in the regulation of various physiological processes in plants, including stomatal dynamics and density, organogenesis, root length and density, cell death, differentiation of vascular tissue, and seed germination [12,13,14].

Plant-microbe interactions are characterized by their dynamic and continuous nature, encompassing both pathogenic and mutualistic relationships. These interactions involve the exchange of signals through distinct molecules produced by the host plant, microbes, or both. Membrane-bound receptor-like kinases play a critical role as receptors in these interactions, facilitating microbe-specific responses through signal transduction. In the context of pathogen infection, the host plant activates defense responses to counteract the invading pathogens. Substantial evidence suggests the involvement of CRKs in plant-pathogen interactions.

Previous studies have demonstrated the significant impact of overexpressing certain cysteine-rich receptor-like kinases on plant resistance against specific pathogens. For instance, *Arabidopsis CRK5*, *CRK6*, *CRK36*, and *CRK45* [9,15,16] have been shown to enhance resistance to *Pseudomonas syringae* by rapidly inducing the expression of defense genes and promoting the production of reactive oxygen species (ROS) [17,18,19]. In *Triticum aestivum*, the overexpression of *TaCRK2* was found to slow down the penetration and intercellular growth of *Zymoseptoria tritici* [20], and *TaCRK2* also exhibited resistance against *Puccinia triticina* infection while positively regulating the hypersensitive reaction (HR) cell death process induced by the pathogen [21].

Furthermore, *TaCRK-7A* was shown to directly inhibit the growth of *Fusarium pseudograminearum* and confer *Fusarium* Crown Rot (FCR) resistance in wheat by promoting the expression of defense genes associated with the jasmonate pathway [22]. Manipulation of the *CaCRK5* expression in *Nicotiana benthamiana* and *Capsicum annum* was found to modulate resistance against *Ralstonia solanacearum* [16], while *GbCRK18* provided resistance to *Verticillium* wilt in *Gossypium barbadense* [7]. The molecular evidence and crystal structure of Gnk2 revealed the importance of fungal mannose binding to specific residues (asparagine-11, arginine-93, and glutamate-104) in Gnk2 for its antifungal activity [5]. These studies collectively highlight the role of CRKs in enhancing plant defense mechanisms against various pathogens and provide insights into their molecular interactions.

The interaction between legumes and rhizobia initiates in the rhizosphere through the exchange of molecular signals between the host’s root hairs and the bacteria. The recognition process during this symbiotic relationship involves crucial molecules, namely, plant-derived isoflavonoids and bacterial-derived Nod factors. These signals play a role in suppressing plant defenses and enabling bacterial access to the epidermal root hairs and cortical cells of the host. This access is facilitated by the formation of a tubular infection thread, guiding the rhizobia towards the division of cortical cells. As a result, a nodule primordium is formed, which eventually gives rise to nodules containing symbiosomes-specialized cell organelles inclosing nitrogen-fixing bacteroids [23,24].

The establishment of symbiosis involves the temporary suppression of defense responses, which is crucial for symbiosome development and bacterial differentiation. Through the study of legume mutants, researchers have identified several host genes contributing to this suppression, including *Medicago SymCRK*, *regulator of symbiosome differentiation* (*RSD*), *defective in nitrogen fixation 2* (*DNF2*), and *nodules with activated defense 1* (*NAD1*) [25,26,27,28]. It has been demonstrated that *DNF2* and *SymCRK* affect defense signaling through the ethylene pathway, while *NAD1* regulates immune responses in nodules via the CDPK-Rboh signaling axis [26,29]. Additionally, a recent genetic study conducted on *Aeschynomene evenia* revealed the requirement of *AeCRK* for triggering both root and stem nodulation [30].

In the context of *Phaseolus vulgaris* L. (common bean), our previous transcriptomic analysis identified several upregulated *CRK* genes in the roots colonized by rhizobia. Among the nine *CRK* genes identified, five were common genes expressed under both mycorrhizal and rhizobial symbiosis conditions, while the remaining four genes *CRK8*, *CRK12*, *CRK20*, and *CRK42* were unique genes expressed exclusively under nodulated conditions. Notably, the upregulation of the *CRK12* gene was particularly significant, as observed in the study by Quezada et al. [10].

Our objective in this study was to conduct a comprehensive functional analysis of the *CRK12* gene in the grain legume *Phaseolus vulgaris*. To achieve this, we employed RNA interference (RNAi) to downregulate and overexpress the *CRK12* gene in transgenic hairy roots of *P. vulgaris*, aiming to investigate its impact on the symbiotic interaction with *Rhizobium*. As a result, the overexpression of *CRK12* genes led to notable changes in root morphology, including increased lateral root and root hair density, as well as longer root hairs. In contrast, silencing of the *CRK12* gene produced contradictory results. During the process of rhizobial colonization, we observed the activity of the *CRK12* promoter in the early stages of symbiosis, specifically at the sites of rhizobia infection units, infection threads, and dividing cortical cells. Quantitative analysis revealed that the overexpression of *CRK12* significantly increased the number of rhizobial infection units and nodule primordia. Moreover, at later stages, these roots exhibited a hypernodulation phenotype compared to the control lines. Conversely, *CRK12*-RNAi roots displayed a phenotype that was contrary to the overexpression lines. Additionally, the ectopic expression of *CRK12* resulted in delayed nodule senescence. Taken together, our findings suggest that *CRK12*, a membrane receptor kinase, is a novel regulator of *Phaseolus vulgaris-Rhizobium tropici* symbiosis.

## 2. Results

### 2.1. Structure of CRK12 Gene and Its Expression

**Figure 1:**
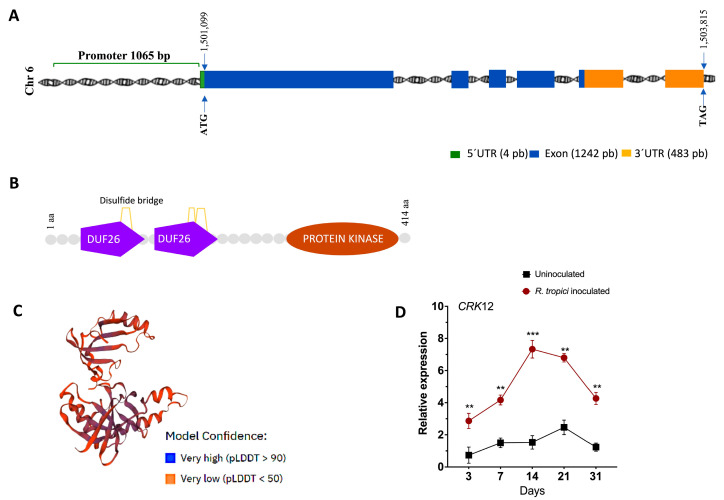
Structure and expression of the *Phaseolus CRK12* gene. (**A**) The gene structure was retrieved from the Phytozome *Phaseolus vulgaris* v2.1 genome database. (**B**) The domain structure was determined based on the ExPASy PROSITE online tool. (**C**) Three-dimensional (3D) protein structure alignment of *CRK12* constructed by a homology-modelling server. (**D**) Quantitative RT–PCR analysis of roots of *P. vulgaris* inoculated with *R. tropici*. Statistical significance was determined using an unpaired two-tailed Student’s *t* test (**, *p* < 0.01; ***, *p* < 0.001), and the data are presented as the means ± SDs. The data shown were obtained from three biological replications (*n* > 9).

The *Phaseolus CRK12* gene sequence was identified in Phytozome 13 database for gene structure studies. The *CRK12* gene is located on chromosome 6, and its gene structure revealed 5 exons and 4 introns in the CDS region and one intron in the 3′ UTR. The mature transcript length was 1729 bp, the CDS was 1242 bp, the 5′ UTR was 4 bp, and the 3′ UTR was 483 bp (Figure 1A). The *CRK12* encodes 413 amino acids with 2 DUF26 domains composed of 2 alpha-helices and a five-stranded beta-sheet, which forms a compact single-domain architecture with an alpha + beta-fold. The DUF26 domain contains a C-X(8)-C-X(2)-C motif, and its structure predicted by the ExPASy PROSITE tool shows cysteine residues form three intramolecular disulfide bridges: C1-C5, C2-C3, and C4-C6 (Figure 1B,C) [31].

We next sought to determine the temporal expression of *CRK12* in wild-type *P. vulgaris* roots. We inoculated the roots with *R. tropici* and monitored *CRK12* expression at different time points: during early signaling (3 dpi); in nodule primordia (7 dpi); and in young, mature, and senescent nodules (14, 21, and 31 dpi, respectively). A significant surge in *CRK12* transcript abundance was observed in all *R. tropic* inoculated root tissues compared to uninoculated root tissues at all the measured time points (Figure 1D). *CRK12* expression was strongly expressed at 3 and 7 dpi, and maximum expression was observed at 14 dpi in the roots. These results indicate that the *CRK12* gene is temporally expressed from the establishment of the nodule to senescence in *P. vulgaris*.

### 2.2. Spatiotemporal Promoter Expression Analysis and Protein Subcellular Localization CRK12

**Figure 2:**
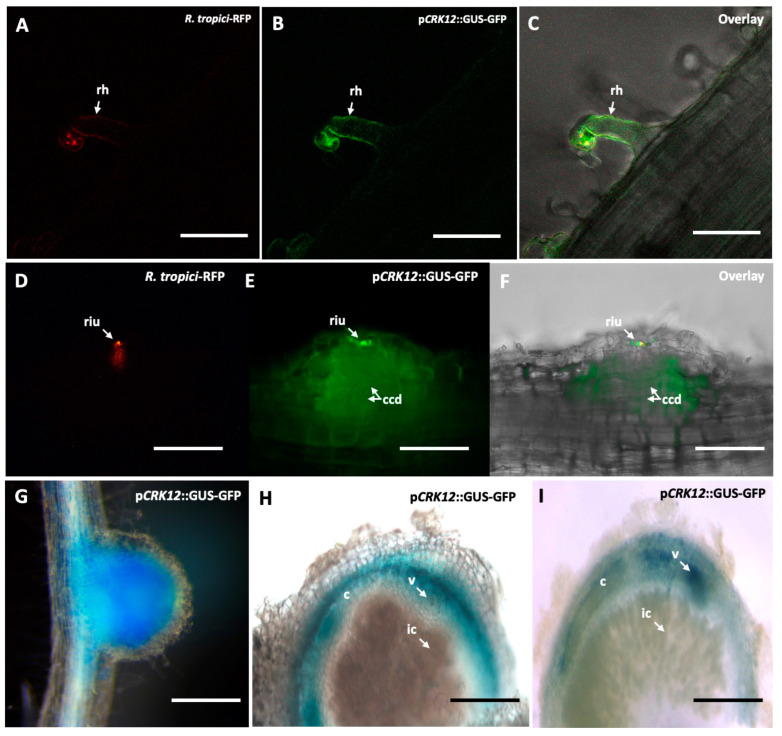
Spatiotemporal expression patterns of the *CRK12* promoter in *P. vulgaris* roots. *Rhizobium tropici* CIAT 899 expressing the RFP marker was inoculated into transgenic hairy roots expressing a p*CRK12*::GUS-GFP construct, and observations were recorded using a Confocal fluorescence microscope at 3 dpi. (**A**) Root hair showing *R. tropici* (RFP marker) infection (**B**) p*CRK12* activity detected as GFP fluorescence and (**C**) overlay. Image of a transgenic root at 6 dpi showing nodule primordia with (**D**) RFP fluorescence at the site of ‘riu’, (**E**) GFP expression at ‘riu’ and ‘ccd’, and (**F**) overlay. The transgenic roots were inoculated with the wild-type *R. tropici* CIAT 899 and assessed to understand the *CRK12* promoter activity in developing nodules using a GUS assay. Representative image of (**G**) a 14-day-old young nodule, nodule sections showing GUS in (**H**) a 21-day-old mature nodule and (**I**) a 28-day-old senescent nodule. dpi, days post-inoculation; rh, root hai; riu, *Rhizobium* infection unit; ccd, cortical cell division; c, cortex; v, vasculature; ic, infected cell. Bars: (**A**–**F**) 20 µm; (**G**) 500 µm; and (**H**,**I**) 1 mm.

To demonstrate spaciotemporal expression of *CRK12* promoter activity in *P. vulgaris* roots under rhizobial symbiosis conditions, we identified and isolated the sequence 1044 bp upstream of the *CRK12* start codon. This isolated promoter fragment was cloned and ligated to drive the expression of two reporters, GUS and GFP, within the vector pBGWFS7.0 [32]. The resultant pBGWFS7.0/*pCRK12*::GUS-GFP binary vector was transformed into the *Agrobacterium rhizogenes* strain K599, which in turn was utilized to generate transgenic hairy roots in *P. vulgaris*. We observed that the transgenic roots at 3 days post-inoculation (dpi) with *R. tropici* (strain CIAT899-RFP) showed *CRK12*-driven GFP expression at the site of rhizobia infection in root hair cells (Figure 2A–C). No such GFP expression was seen in the root hair cells of uninoculated transgenic roots (Supplementary Figure S1). GFP expression intensified during nodule primordium formation specifically at the site of the *Rhizobium* infection and in the dividing cortical cells of the nodule (Figure 2D–F). Transgenic roots containing 14-, 21-, and 28-day-old nodules were assayed for the histochemical localization of GUS. Fourteen-day-old young nodules presented an intense GUS color (Figure 2G). The sections show that *CRK12* was most active in the inner cortical cells and vasculature of the 21-day-old mature nodules (Figure 2H). Upon the onset of nodule senescence (28 days old), GUS activity was decreased in the nodules (Figure 2I). Together, our results showed that at the early stages of nodule development, the *CRK12* promoter was active in ITs, dividing cortical cells of nodule primordia. Strong *CRK12* promoter activity was observed in the young nodules, whereas in the mature nodules, the activity was restricted to the inner cortical cells and vasculature of the mature and senescent nodules of *P. vulgaris*.

**Figure 3:**
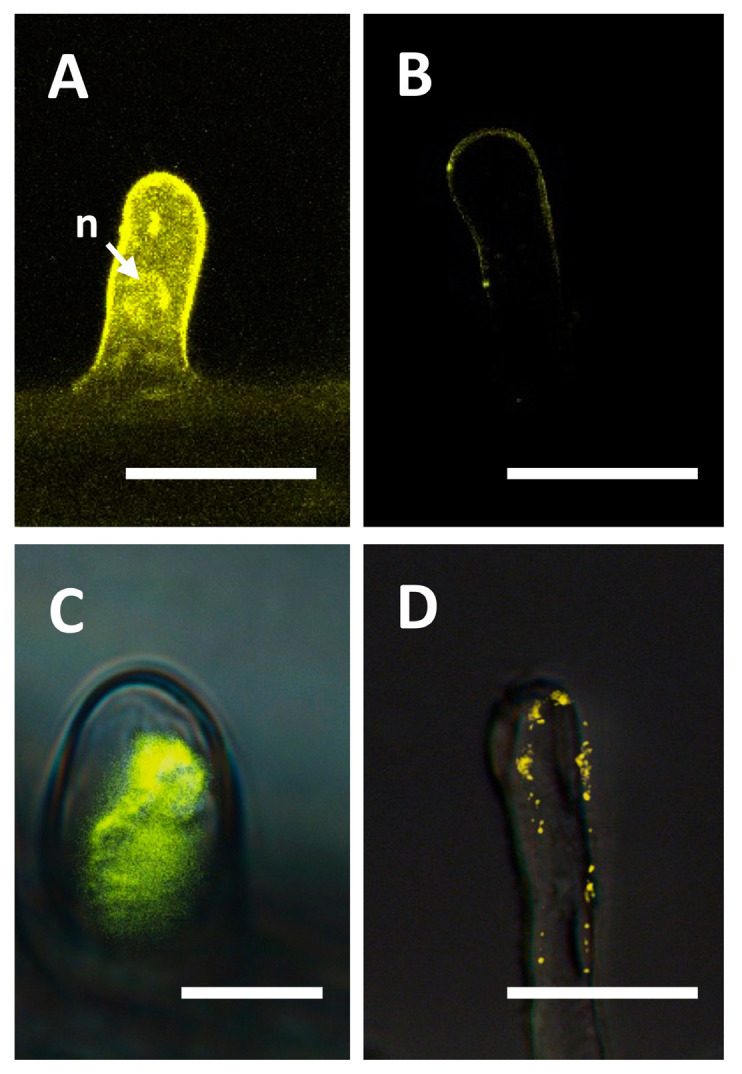
Subcellular localization of *Phaseolus CRK12*. The ORF of *PvCRK12* was cloned into pEarleyGate104 to construct an N-terminal YFP, which was fused and transformed into *P. vulgaris* hairy roots to determine the subcellular localization of the protein. The images were obtained with a confocal microscope equipped with a digital camera. (**A**) The nonfused 35S-YFP control construct localizes to the cytoplasm and nuclei. (**B**) The YFP-CRK12 construct exhibits plasma membrane localization in growing root hairs. Plasmolysis was induced in *P. vulgaris* root hair cells by treatment with 150 mM NaCl for 12 min before imaging; (**C**) control, and (**D**) YFP-CRK12. Bars = 10 µm. n, nucleus.

To investigate the subcellular localization of the CRK12 protein, a pEarleyGate104 vector was used for a transient expression of the CRK12 protein fused to yellow fluorescent protein (YFP). The confocal images of the *P. vulgaris* hairy roots expressing YFP-CRK12 showed that CRK12 was localized to the plasma membrane of the root hair cells (Figure 3B), and hairy roots expressing non-fused YFP were used as controls. As predicted, unfused YFP was observed in both the cytoplasm and nuclei of the root hair cells (Figure 3A). NaCl (250 mM)-induced plasmolysis further confirmed the association of fluorescence with the plasma membrane (Figure 3D). In contrast, plasmolysis of control root hairs showed that YFP fluorescence remained in the cytoplasm (Figure 3C). Simultaneously, we analyzed the subcellular localization using an in silico tool. For this, the full protein sequence of CRK12 was submitted to the protein subcellular localization prediction tool WoLF PSORT (https://www.genscript.com/wolf-psort.html?src=leftbar, accessed on 15 March 2022). As anticipated, at the subcellular level, the CRK12 protein was targeted to the plasma membrane.

### 2.3. CRK12 Alter Root and Root Hair Morphology

**Figure 4:**
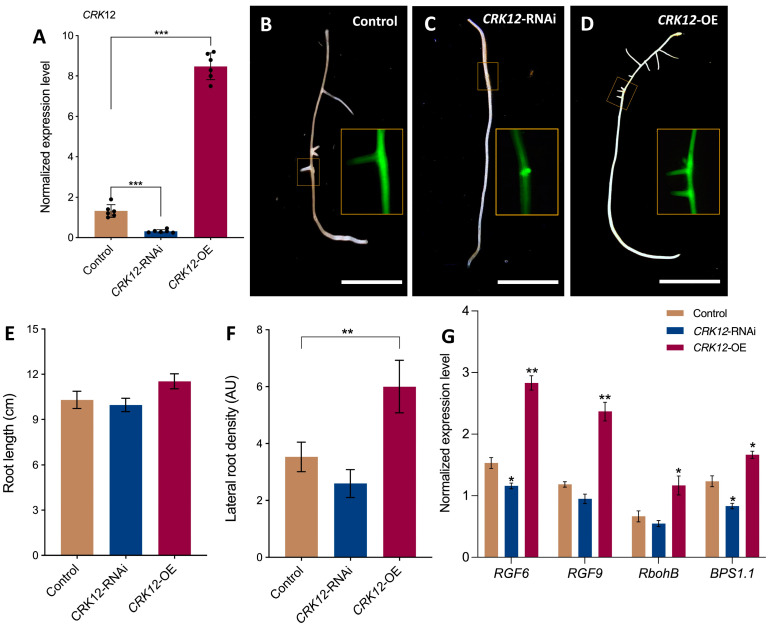
Quantitative RT–PCR of *CRK12* and phenotypes of transgenic roots. (**A**) Transcript abundance of *CRK12* in the *CRK12*-RNAi, *CRK12*-OE and control hairy roots at one-week post-emergence (wpe), as measured using quantitative RT–PCR. Phenotypes of the length and lateral root numbers of (**B**) control, (**C**) *CRK12*-RNAi, and (**D**) *CRK12*-OE transgenic hairy roots at one wpe. The inset images show the transgenic nature of hairy roots expressing a visible marker, eGFP. Quantitative analysis of (**E**) primary root length and (**F**) lateral root density in *CRK12*-RNAi, *CRK12*-OE and control hairy roots. (**G**) Expression levels of root meristem regulatory genes, viz., *RGF6*, *RGF9*, *RbohB* and *BPS1.1*, in *CRK12*-RNAi, *CRK12*-OE and control hairy roots, as assessed by quantitative RT–PCR. The statistical significance of differences between the control group and *CRK12*-RNAi or *CRK12*-OE group was determined using an unpaired two-tailed Student’s *t* test (* *p* < 0.05; ** *p* < 0.01; *** *p* < 0.001). The error bars represent the means ± standard errors of the means (SEM). The data shown were obtained from three biological replications (*n* > 6 for (**A**); *n* > 27 for (**D**,**E**); *n* > 9 for (**F**)). Bars: (**B**–**D**) 1 cm.

To investigate the function of *CRK12*, we generated *P. vulgaris* with transgenic hairy roots expressing *CRK12*-RNAi and *CRK12-OE* to observe the root and nodule phenotypes under symbiotic conditions. The non-conserved sequence of *CRK12* or complete coding sequence of *CRK12* was isolated from fresh *P. vulgaris* cDNA and cloned into a pK7GWIWG2D(II) and pH7WG2D.1 binary vector downstream of the constitutive 35S promoter, respectively. *Agrobacterium rhizogenes* K599 harboring a 35S-promoter::*CRK12*-RNAi (*CRK12*-RNAi) or 35S-promoter::*CRK12* (*CRK12*-OE) was used to generate the transgenic hairy roots. The quantitative RT–PCR results showed (Figure 4A) a 0.76-fold lesser and 5.44-fold greater *CRK12* transcript abundance in the *CRK12*-RNAi and *CRK12*-OE roots, respectively, compared to the control roots (those expressing the empty pH7WG2D.1 vector), demonstrating transcript downregulation and overexpression in respective roots of the *P. vulgaris* transgenic roots.

The phenotypes of the *CRK12*-RNAi, *CRK12*-OE transgenic and control hairy roots were analyzed at 7 days post-emergence. A pK7GWIWG2D(II)-RNAi vector and pH7WG2D.1 vectors expressing a visible marker, eGFP (Figure 4C,D), were used to select the transgenic roots of EV control and *CRK12*-RNAi and *CRK12*-OE composite transgenic plants (which had transgenic hairy roots but wild-type shoots). The primary root length had marginally decreased in *CRK12*-RNAi and slightly, but not significantly, increased in *CRK12*-OE plants compared to controls (Figure 4C–E). However, the density of lateral roots was found marginally decreased in *CRK12*-RNAi and significantly increased in the *CRK12*-OE plants relative to the controls (Figure 4F). Quantitative RT–PCR analysis of these roots showed a significant surge in the abundance of transcripts of root meristem regulatory genes such as the root meristem growth factor-like6 (*RGF6*) and *RGF9,* and respiratory burst oxidase homologues, *RbohB* and *BPS1.1* (*Bypass 1.1*), in the transgenic roots, in which *CRK12* was overexpressed and a transcript downregulation in *CRK12* silenced roots was recorded compared to the control roots (Figure 4G). These results indicated that the overexpression of *CRK12* increased the lateral root numbers, and which could be justified by the abundance of transcripts of genes related to lateral root development in *P. vulgaris*.

**Figure 5:**
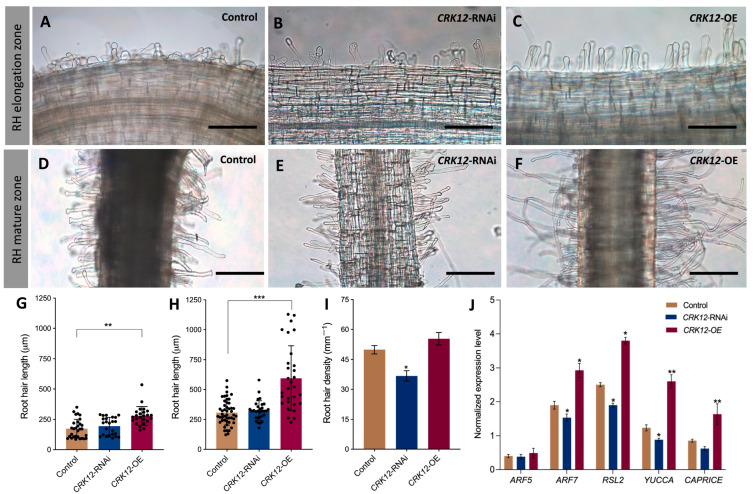
Qualitative and quantitative analysis of root hair morphology of transgenic roots. Root hair morphology in the (**A**–**C**) RH elongation zone and (**D**–**F**) RH mature zone in the control, *CRK12*-RNAi and *CRK12*-OE transgenic hairy roots at 10 dpe. Quantitative analysis of root hair length at the (**G**) RH elongation zone and (**H**) RH mature zone in *CRK12*-OE, *CRK12*-RNAi and control hairy roots. (**I**) Quantitative analysis of root hair density in the RH mature zone. (**J**) Expression levels of genes that regulate the growth and elongation of root hairs, viz., *ARF5*, *ARF7*, *RSL2*, *YUCCA* and *CAPRICE*, in *CRK12*-RNAi, *CRK12*-OE and control hairy roots, as measured by quantitative RT–PCR. The statistical significance of differences between control group and *CRK12*-RNAi or *CRK12*-OE group was determined using an unpaired two-tailed Student’s *t* test (* *p* < 0.05; ** *p* < 0.01; *** *p* < 0.001). The error bars represent the means ± standard errors of the means (SEM). The data shown were obtained from three biological replications (*n* > 27 for (**E**–**G**); *n* > 9 for (**H**)). Bars = 500 µm.

Next, the root hair morphology of the transgenic roots at 10 days post-emergence was analyzed. Observations via light microscopy revealed a decrease in the density of root hairs both in root hair elongation (Figure 5A–C) and maturation zone (Figure 5D–F) of *CRK12*-RNAi roots and the contrary was true for the *CRK12*-OE plants compared to the controls. In the elongation and mature zones of *CRK12*-OE roots, the root hairs exhibited a range of lengths, from 279 µm to 594 µm. In comparison, the control group had root hairs measuring 174 µm in the elongation zone and 307 µm in the mature zone, which were comparable to the measurements of *CRK12*-RNAi root hairs, specifically 195 µm and 325 µm, respectively (Figure 5G,H). However, the root hairs were slightly denser (but not statistically different) on the *CRK12*-OE roots than control roots. In contrast, the density of *CRK12*-RNAi root hairs was significantly lower than that of the control (Figure 5I). Subsequently, several genes, viz., *auxin response factor 5 and 7* (*ARF5*, *ARF7*), *RHD6* (*root hair defective 6*)-*like 2* (*RSL2*), *YUCCA* and *CAPRICE*, which regulate the growth and elongation of root hairs, were analyzed. The subsequent qPCR results showed that *ARF7*, *RSL2*, *YUCCA* and *CAPRICE* transcripts increased significantly in the *CRK12*-OE roots relative to the *CRK12*-RNAi and control roots (Figure 5J). Together, these results suggested that the overexpression of *CRK12* increased root hair length and the expression of root hair regulatory genes.

### 2.4. CRK12 Regulates Nodule Numbers and Infection Units in P. vulgaris

**Figure 6:**
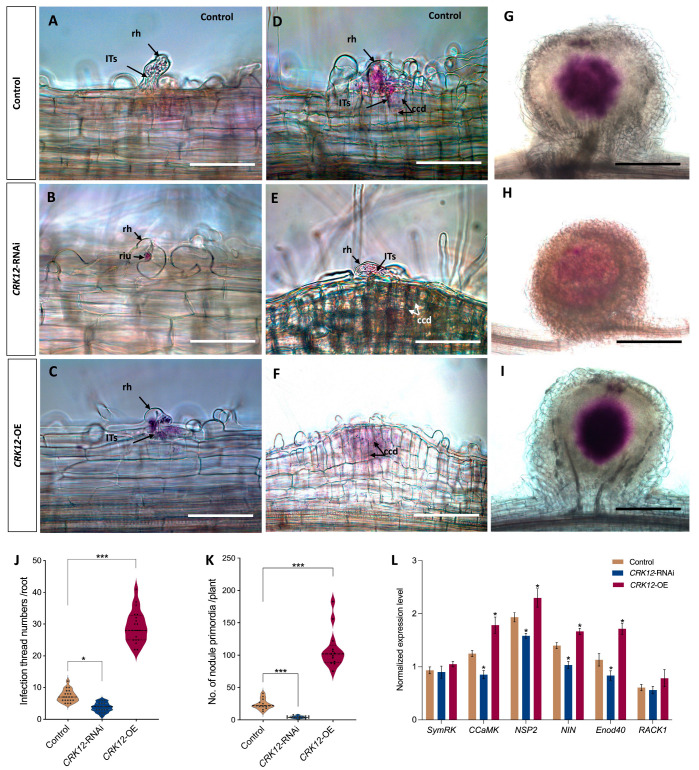
Response to rhizobia infection of *CRK12* transgenic roots. The control, *CRK12*-RNAi and *CRK12*-OE transgenic roots were inoculated with *Rhizobium tropici* CIAT 899 expressing the GUS reporter, and observations were recorded. GUS-assayed roots showing rhizobial infection units at 7 dpi on (**A**) control, (**B**) *CRK12*-RNAi and (**C**) *CRK12*-OE transgenic roots. The roots showing characteristic (**D**–**F**) infection threads and dividing cortical cells in control and *CRK12*-OE transgenic roots. Similarly, GUS-stained young nodules in (**G**) control, (**H**) *CRK12*-RNAi and (**I**) *CRK12*-OE transgenic roots. The data depicted in the violin plot shows (**J**) infection thread numbers per transgenic root and (**K**) the average number of nodule primordia per plant. (**L**) Expression levels of key early signaling genes viz., *SymRK*, *CCaMK*, *NIN*, *Nsp2*, *Enod40* and *RACK1*, in *CRK12*-RNAi, *CRK12*-OE and control hairy roots, as measured by quantitative RT–PCR. The statistical significance of differences between control group and *CRK12*-RNAi or *CRK12*-OE group was determined using an unpaired two-tailed Student’s *t* test (* *p* < 0.05; *** *p* < 0.001). The error bars represent the means ± standard errors of the means (SEM). The data shown were obtained from three biological replications (*n* > 30). dpi, days post-inoculation; riu, rhizobium infection unit; rh, root hair; IT, infection thread; ccd, cortical cell division. Bars: (**A**–**D**) 20 µm; (**E**,**F**) 50 µm; and (**G**–**I**) 200 µm.

To assess the *CRK12* overexpression and down-regulation effect on nodulation, we first inoculated the composite transgenic plants with *R. tropici* CIAT 899 expressing a *GUS* reporter [33]. Periodically, the roots were assayed for GUS and analyzed to determine the symbiosis phenotype. After one week of CIAT 899 inoculation, the frequency of infection events on the *CRK12*-RNAi revealed to be lower (3.8/plant) and *CRK12*-OE roots was higher (29/plant) than that on the control roots (7.4/plant; Figure 6A–C,J). Light microscopy observations in *CRK12*-OE and *CRK12*-RNAi revealed that the phenotype of early nodule development processes, such as the root hair infection thread progression and cortical cell division of nodule primordium, were like those of the control roots (Figure 6C–F).

Next, we compared the number of nodule primordia in the *CRK12*-RNAi and *CRK12*-overexpression to that of control roots. Significantly higher number of nodule primordia (108/plant) were observed in the *CRK12*-OE roots than in the control roots (24/plant) at 10 days post-inoculation in the contrary, *CRK12* downregulation led to a highly significant reduction in nodule primordia (5/plant; Figure 6K). All the young nodules of the control and *CRK12*-OE roots were colonized successfully with rhizobia, whereas the *CRK12*-RNAi show poor rhizobia density (Figure 6G–I).

To determine whether this phenotype is associated with changes in the expression of genes involved in early rhizobial signaling, we measured the expression levels of some of the key early signaling genes, such as *SymRK* (*symbiosis receptor kinase*), *CCaMK* (*calcium-calmodulin kinase*), NIN (*nodule inception*), *Nsp2* (*nodule signaling pathway 2*), *Enod40* (*early nodulin 40*) and *RACK1* (*receptor for activated C kinase 1*). Based on the quantitative RT–PCR results, *CCaMK*, *NIN*, *Nsp2* and *Enod40* showed a significant surge in transcript levels in the *CRK12*-OE transgenic roots compared with the *CRK12*-RNAi and control roots, whereas *SymRK* and *RACK1* showed slightly higher expression in the *CRK12*-OE roots relative to controls (Figure 6L). Together, our data indicate that *CRK12* functions during the early stages of nodule formation and development, which is reflected in terms of increased rhizobial infection units, nodule primordial numbers and increased expression of early signaling genes in *P. vulgaris*.

### 2.5. CRK12 Overexpression Results in Hypernodulation in P. vulgaris Transgenic Roots

**Figure 7:**
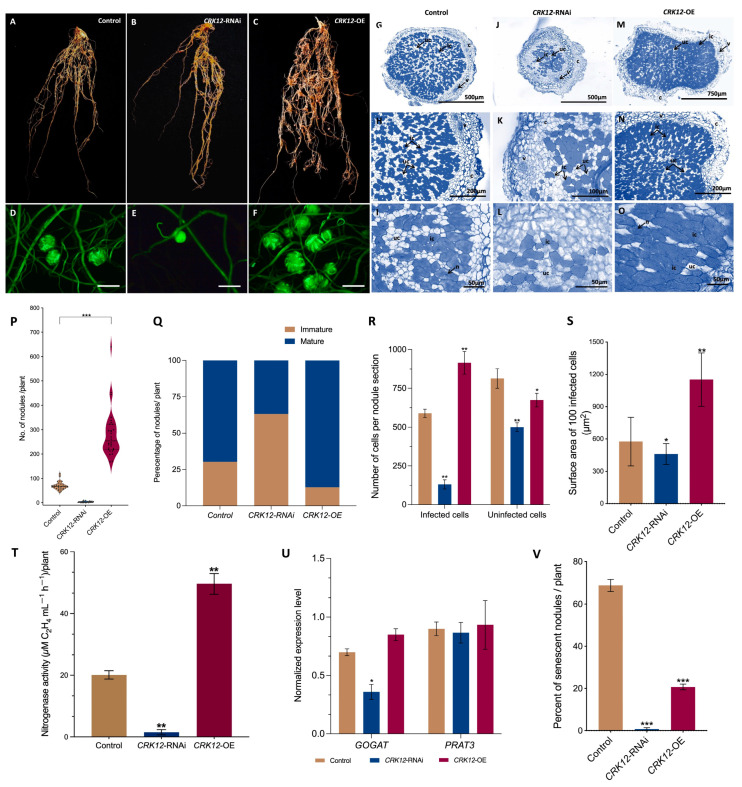
Nodule phenotype and RT–qPCR analysis of *CRK12* transgenic roots. Representative images of transgenic whole root system of (**A**) control, (**B**) *CRK12*-RNAi and (**C**) *CRK12*-OE showing nodules numbers at 21 days post inoculation with *Rhizobium tropici* CIAT899. GFP expression of the nodulated transgenic roots of the (**D**) control, (**E**) *CRK12*-RNAi and (**F**) *CRK12*-OE under a fluorescence stereomicroscope. Toluidine blue-stained transverse sections of (**G**–**I**) control and (**J**–**L**) *CRK12*-RNAi, (**M**–**O**) *CRK12*-OE nodules revealing structural features as shown in light micrographs. (**P**) Quantitative analysis showing the average number of mature nodules per plant. (**Q**) Percentage of nodules per control, *CRK12*-RNAi and *CRK12*-OE composite plants at 21 days post inoculation. (**R**) Average number of infected and uninfected cells, and (**S**) Infected cell-surface area of transgenic nodules. (**T**) Nitrogenase activity in control, *CRK12*-RNAi and *CRK12*-OE nodulated transgenic roots inoculated with at 21 dpi, as determined by an acetylene reduction assay. (**U**) Expression levels of *GOGAT* and *PRAT3* genes in the control and *CRK12* transgenic nodules. Quantitative RT–PCR was performed using freshly isolated RNA from 21-day old mature nodules of control and *CRK12*-OE. Error bars represent the means ± standard error of the mean (SEM). (**V**) Percentage of senescent nodules per control and *CRK12*-OE composite plants at 35 days post inoculation. The statistical significance of differences between control group and *CRK12*-RNAi or *CRK12*-OE group was determined using an unpaired two-tailed Student’s *t* test (* *p* < 0.05; ** *p* < 0.01; *** *p* < 0.001). The error bars represent the means ± standard errors of the means (SEM). The data shown were obtained from three biological replicates (*n* > 30). dpi, days post-inoculation; uc, uninfected cell; ic, infected cell; c, cortex; n, nucleus; v, vascular bundles. Bars: (**D**–**F**) 2 mm.

While we were trying to identify the impact of the *CRK12* transcript down-regulation on root nodule symbiosis, at 21-day post inoculation we found that the nodule numbers remained critically low. The *CRK12*-RNAi transgenic roots exhibited fewer number of nodules and were remained to be juvenile/primordial implying their failure to reach to mature nodule stage. In addition, the transgenic *CRK12*-OE roots shows increased nodule numbers compared to control transgenic roots (Figure 7A–F). Furthermore, the quantitative data revealed a 4-fold higher number of nodules in the *CRK12*-OE roots than in the control roots. The average number of nodules was 71.7 per control plant, 4 per RNAi and 289.3 per *CRK12*-OE plant (Figure 7P). Among them, 87.1 percent of the nodules were mature pink at 21 dpi, and the remaining were immature white nodules in the *CRK12*-OE roots. In control 69.7 percent of the nodules were pink, and 30.3 percent were white. Nevertheless, the *CRK12*-RNAi roots show 36.8 and 63.2 percent pink and white nodules, respectively (Figure 7Q).

The transverse section of mature control nodules (Figure 7G–I) depicted typical histological characteristics of a determinate nodule, such as the outer and inner cortex, nodule vasculature, and nodule core [34]. The nodule core tissue was comprised of infected cells harboring *R. tropici* CIAT 899 and uninfected cells (Figure 7I). On the other hand, the *CRK12*-RNAi nodules were smaller in size. However, the nodule structural details remained comparable to controls (Figure 7J–O). The most interesting detail was in the highly reduced number of infected cells in the *CRK12*-RNAi nodules (Figure 7L). The *CRK12*-OE nodules displayed characteristics similar to control (Figure 7G,H,M,N) except the nodule core showing a significant increase in infected cell density compared to the control and *CRK12*-RNAi (Figure 7R). On the contrary, the uninfected cells numbers were fewer than the control. Additionally, the infected cell area in the *CRK12* overexpressed nodules were significantly larger (1151 µm^2^/100 cells) than the control (577 µm^2^/100 cells) and *CRK12*-RNAi (460 µm^2^/100 cells) (Figure 7S).

Subsequently, at 21 dpi, we estimated the nitrogen fixation rate in control, *CRK12*-RNAi and *CRK12*-OE transgenic roots and results showed a significant increase of nitrogen fixation in the *CRK12*-OE nodules compared to controls; at the same time, *CRK12*-RNAi demonstrated highly reduced abilities to fix nitrogen (Figure 7T). Next, we measured key organic nitrogen export-related genes, such as *Gln synthetase* and *glutamate synthase* (*GOGAT*), and *glutamine phosphoribosyl pyrophosphate amidotransferase 3* (*PRAT3*) in the mature nodules. The quantitative RT–PCR results showed similar expression patterns for both *GOGAT* and *PRAT3* in the *CRK12*-OE and control nodules at 21 dpi, in *CRK12*-RNAi roots the *GOGAT* significantly decreased compared to control (Figure 7U). At 35 dpi, the nodule morphology showed that 70% of control root nodules were senescent, and only 21% of the *CRK12*-overexpressing nodules were senescent. These results indicate the prolonged nitrogen fixing capabilities of the *CRK12*-overexpressing root nodules (Figure 7V).

Together, these data suggest that the transgenic roots that expressed the *CRK12*-RNAi vector severely affected root nodule numbers and their nitrogen fixing abilities. On the contrary, overexpression of *CRK12* showed a phenotype with increased nodules numbers and infected cell density and size. Furthermore, these overexpressed nodules fixed more nitrogen and the presence of key nitrogen export genes in these nodules confirmed the function of these nodules.

## 3. Discussion

Receptor-like kinases (RLKs) possess distinct extracellular domains that enable the recognition of different ligands and facilitate the transduction of various extracellular signals, including those involved in symbiosis [35,36]. Symbiosis is initiated by legumes and rhizobia exchanging signaling molecules as part of a bidirectional communication process. In response to host-secreted flavones or isoflavones, rhizobia synthesize and discharge nod factors, also known as lipo-chitooligosaccharides (LCOs) [37]. These LCOs are perceived by LysM-type plasma membrane receptors such as *NFR1*, *NFR5*, and *NFRe* in *Lotus japonicus*; among them, both *NFR1* and *NFR5* were essential for the nod factor signaling [38,39], whereas *NFRe* may increase signaling in root epidermal cells [40]. Downstream of LysM-type receptors, a cascade of symbiotic signaling genes and transcription factors (TFs) function in triggering infection thread formation, nodule organogenesis and other processes in legume roots [41].

The largest group of plant RLKs consists of cysteine-rich receptor kinases or proteins that possess the DUF26 domain. However, the biological functions of these RLKs in plant symbiotic interactions have been relatively understudied. Earlier investigations in *Medicago truncatula* have demonstrated that mutations in *symCRK* result in the formation of nodules exhibiting defense-like reactions, bacterial death, and ultimately an inability to fix nitrogen [26,42,43]. In a previous study focused on *P. vulgaris*, it was indicated that potential *PvCRK* genes may play a role in regulating these diverse symbiotic interactions [10]. Additionally, more recent research had discovered that *AeCRK* is crucial for initiating root and stem nodulation in *Aeschynomene evenia* [30].

The main outcome of our preliminary investigations in *P. vulgaris* led us to perform a functional characterization of *CRK12* in the current study. This investigation aimed to elucidate the specific role of CRK12 in the interactions between *P. vulgaris* and *Rhizobium tropici*. CRK12 in *Phaseolus* is characterized by two DUF26 domains that contain a C-X(8)-C-X(2)-C motif similar to other CRKs [10]. Although the precise function of this domain remained unknown, previous reports suggested its potential involvement in redox regulation and protein–protein interactions [44,45]. Interestingly, the temporal expression patterns of CRK12 in plants inoculated with *Rhizobium* symbionts showed an increase in transcript levels at all stages of symbiosis. The analysis of *cis*-elements in the regulatory region of *CRK12* indicated the absence of symbiosis-specific transcription factors [10]. However, an abundance of transcription factors involved in phytohormone regulation was identified, suggesting the presence of a potential signaling mechanism [46,47] (Lin et al., 2020; Li et al., 2022). To further investigate this, we examined the expression of the CRK12 gene promoter, which yielded intriguing results. The *CRK12* promoter exhibited expression in *Rhizobium* infection units, infection threads, dividing cortical cells, inner cortex, and vasculature of mature nodules.

The roles of *CRK*s in growth and developmental aspects of plants have been previously characterized [6]. Herein, the overexpression of *CRK12* resulted in an increased density of lateral roots as well as root hairs, and root hairs grew longer both in the root hair elongation and in the maturation zones in comparison to the controls. Conversely, when *CRK12* expression was suppressed using RNA interference (RNAi), we observed a contrasting phenotype in the roots and root hairs, thereby reinforcing the significance of this gene in the development of roots and root hairs. Interestingly, our findings differ from previous studies on *Arabidopsis CRK28*, *CRK29*, and *CRK42* mutants, where mutations in *CRK* genes resulted in longer primary roots and denser lateral roots [6,13].

To gain insights into the underlying mechanism behind the altered root phenotype, we conducted transcript analysis of key genes involved in regulating root hair length, including auxin responsive factors (*ARF5*, *ARF7*) [48] and the auxin biosynthesis gene *YUCCA* [49]. We found significantly higher expression levels of these genes in *CRK12*-overexpressing (*CRK12*-OE) roots compared to the *CRK12*-RNAi and control roots. Additionally, transcripts related to root growth regulation, such as *RGF6*, *RGF9* [50], *RbohB* [51], and *BPS1.1* [52], were also induced in *CRK12*-OE roots. These findings suggested a potential mechanism underlying the observed root and root hair phenotypes. It is important to note that root hairs serve as entry points for rhizobia, and an increased density of root hairs could enhance the opportunity for symbiotic interactions with these microorganisms.

Previous studies have demonstrated the involvement of *CRKs* in immune responses during plant–pathogen interactions [17,53]. These CRKs perceive signals through extracellular, transmembrane, and intracellular domains, leading to the activation of MAPK pathways and subsequent gene transcription [54]. In *Medicago truncatula*, the participation of SymCRK, a cysteine-rich receptor-like kinase, has been reported in mutualistic interactions, such as symbiosis [43]. In this study, we focused on investigating the effects of the silencing and overexpression of *CRK12* on rhizobial nodule symbiosis (RNS). In *Phaseolus* plants, overexpression of *CRK12* resulted in a remarkable rise in the occurrence of infection events, with 108 events per plant, which was significantly higher compared to the controls with only 24 events per plant. This increase was also observed in the number of nodules, as *CRK12*-OE led to 289.3 nodules in the roots, representing a fourfold increase compared to the control group which had 71.7 nodules. Conversely, the silencing of *CRK12* resulted in a notable decrease in both infection events (5 events per plant) and nodule numbers (4 nodules per plant). Furthermore, the few nodules observed in the *CRK12*-RNAi plants displayed similar anatomical characteristics to the control but had very few infected cells. In contrast, histological observations of *CRK12*-overexpressing (*CRK12*-OE) nodules revealed an increase in both the number and size of infected cells. These findings are consistent with the nitrogen-fixing abilities exhibited by both *CRK*-RNAi and *CRK*-OE nodules. Previous reports involving SymCRK in *M. truncatula* mutants did not show a significant change in nodule numbers however, most of the nodules developed were nonfunctional necrotic nodules [26]. Further, the investigation of *Aeschynomene evenia*’s stem and root nodule development indicated that *AeCRK*, in conjunction with other symbiotic pathway genes, was essential for the process.

In *Medicago*, the overexpression of a lectin-like receptor kinase (LecRK), a potential rhizobial lipochitooligosaccharide-binding RLK, has been shown to increase nodule numbers [55]. In the present study, the observed increase in infection events and nodule numbers upon overexpression of *CRK12*, as well as the contrasting effect when *CRK12* transcript was downregulated, suggest a potential role of *CRK12* as a receptor for rhizobial nod factors. Furthermore, in *Phaseolus* plants, the overexpression of *CRK12* led to the upregulation of *respiratory burst oxidative homologue B* (*RbohB*), resulting in increased levels of reactive oxygen species. Previous studies have reported the involvement of *RbohB* in maintaining symbiosome number, bacteroid size, and nitrogen fixation in *Phaseolus* nodules [56]. Notably, CRKs have been implicated in direct ROS sensing due to the redox regulation possibilities within their extracellular protein domain [6]. Hence, it is plausible to propose that the crosstalk between CRK12 and ROS signaling may contribute to the observed increase in nodule numbers.

In conclusion, our investigations provide compelling evidence of the significant influence exerted by *CRK12* on the development of root hairs and root nodules, as well as nitrogen fixation in *P. vulgaris*. These findings underscore the undeniable role played by *CRK12* in governing the mutualistic association between *R. tropici* and *P. vulgaris*, as downregulation or overexpression of *CRK12* transcripts directly impact these processes. Finally, we suggest that harnessing symbiosis-specific gene(s) like *CRK*s in breeding programs for genetic modification presents exciting opportunities to enhance legume crops, leading to improved nitrogen fixation and supporting more sustainable and productive agricultural practices.

## 4. Materials and Methods

### 4.1. Plant Material and Rhizobium Inoculation

Seeds of *Phaseolus vulgaris* cv. Negro Jamapa obtained from the Instituto de Biotecnología, UNAM, Mexico, were used in this study. The seeds were surface disinfested and germinated, as described previously by Nanjareddy and colleagues [57]. The seedlings were then transplanted into sterile vermiculite in the greenhouse under a 16 h photoperiod at 28 ± 1 °C. The plants were irrigated with Broughton and Dilworth (B&D) [58] nutrient media with limited amounts of nitrogen (2 mM KNO_3_) to promote root nodulation. For the induction of nodules, 1 mL of *Rhizobium tropici* (wild-type strain CIAT899 or that expressing RFP or a GUS reporter) at an OD_600_ dilution of 0.6 was inoculated. Root or nodule tissues were collected at various time points, and the samples were immediately immersed in liquid nitrogen and stored at −80 °C.

### 4.2. Gene and Protein Structure

The exon–intron structure was determined using the genomic sequence of the *CRK12* gene in Phytozome 13. *Phaseolus CRK12* protein domains were predicted by PROSITE (https://prosite.expasy.org/, accessed on 29 April 2022), and their secondary structure was predicted using the Swiss model (http://swissmodel.expasy.org, accessed on 29 April 2022).

### 4.3. Promoter Construction and Composite Plant Production

To analyze the spatiotemporal expression patterns of the *CRK12* promoter during nodulation, a 1044 bp promoter fragment was amplified from *P. vulgaris* genomic DNA using specific primers (Supplementary Table S1) and cloned into a pENTR/D-TOPO vector (Invitrogen, Carlsbad, CA, USA). The Gateway LR reaction was performed between the entry vector pENTR/D-TOPO-*pCRK12* and the destination vector pBGWSF7.0 according to the manufacturer’s (Invitrogen) instructions. The sequence of the resultant pBGWSF7.0-*pCRK12*::GUS-GFP was verified.

To generate the *P. vulgaris* composite transgenic plants (whose hairy roots expressed the recombinant plasmid vector), 2-day-old germinated seedlings were infected with *Agrobacterium rhizogenes* K599 strains carrying the pBGWSF7.0-*pCRK12*::GUS-GFP construct. The empty pBGWSF7.0 vector was used as a control. The plasmid constructs were introduced into *A. rhizogenes* strain K599 separately. All the composite transgenic plants were generated as described by Nanjareddy et al. [57], after which they were transplanted into sterile vermiculite and inoculated with the wild-type *Rhizobium tropici* CIAT 899 or that expressing RFP markers, as previously described by Nanjareddy & colleagues [57].

### 4.4. Subcellular Localization Analysis of CRK12

The open reading frames of *CRK12* were amplified from cDNA freshly prepared from 10-day-old *P. vulgaris* seedlings using the appropriate oligonucleotides (Supplementary Table S1). The amplicon was cloned into a pENTR/D-TOPO vector. The Gateway LR reaction was performed between the entry vector pENTR/D-TOPO-*CRK12* and the destination vector pEarleyGate104 according to the manufacturer’s (Invitrogen) instructions. The N-terminus of the resulting binary vector consisted of fusion of CRK12 and yellow fluorescent protein (YFP). A pEarleyGate104 vector expressing YFP was used as a control.

### 4.5. Cloning of CRK12 Overexpression and Silencing Constructs

The primer pair used (Supplementary Table S1) was designed to amplify the open reading frame of the *CRK12* gene (Phvul.006G006800) from freshly prepared *P. vulgaris* seedling cDNA. The amplified fragment of 1271 bp (1242 bp ORF + 29 bp 3′UTR) was cloned into a pENTR/D-TOPO vector. The Gateway LR reaction was performed between the entry vector pENTR/D-TOPO-*CRK12* and the destination binary vector pH7WG2D.1, which was under the control of the constitutive 35S promoter [32] according to the manufacturer’s instructions (Invitrogen). The sequence of the resultant pH7WG2D.1-*CRK12*-OE was verified. The empty pH7WG2D.1 vector was used as a control in the experiments.

The *CRK12*-RNAi silencing construct was created through the cloning of a non-conserved 280 bp fragment from the 3′UTR of *CRK12*. This fragment was amplified using common bean root cDNA as a template and specific primers (Supplementary Table S1). Subsequently, the fragment was inserted into the pENTR/D-TOPO vector, and the resulting construct was combined with the binary vector pK7GWIWG2D(II) using Gateway Technology (Invitrogen Gateway cloning technology). To ensure the correct orientation of the inserted fragments in the *CRK12*-RNAi construct, PCR and sequencing were conducted for verification. The empty pK7GWIWG2D(II) vector was used as a control in the experiments.

The composite transgenic plants were generated as described above. After removing the wild-type primary root from the composite transgenic plants, we selected the hairy roots under an epifluorescence microscope with a GFP filter with an excitation of 488 nm and an emission fluorescence ranging from 510 to 540 nm. At the same time, the non-transformed roots were removed, transplanted into sterile vermiculite and then inoculated with the *R. tropici* CIAT 899 strain.

### 4.6. Expression Analysis

Total RNA was extracted from frozen tissues using a Spectrum™ Plant Total RNA Kit (Merck KGaA, Darmstadt, Germany) following the manufacturer’s instructions. Contaminant DNA among the RNA samples was eliminated by incubating the samples with RNase-free DNase (1 U µL^−1^) at 37 °C for 15 min. The RNA integrity and concentration were verified via 1% agarose gel electrophoresis and via spectrometry with a NanoDrop^TM^ 2000 instrument, respectively.

Quantitative PCR was performed using an iScript^TM^ One-Step RT–PCR Kit with SYBR^®^ Green following the manufacturer’s (Bio-Rad, Hercules, CA, USA) recommendations in a Bio-Rad iQ^TM^ 5 real-time PCR detection system. Each reaction had included 40 ng of RNA as a template. A control sample without reverse transcriptase was included to confirm the absence of contaminant DNA. Relative gene expression levels were calculated using the formula 2^–ΔCT^, where the cycle threshold value (Δ^CT^) is the CT of the gene of interest minus the CT of the reference gene. The relative expression values, normalized to those of two reference genes (*Phaseolus EF1a*—‘*elongation factor 1-alpha*’ and *IDE—*‘*insulysin*’), were calculated according to the methods of Vandesompele et al. [59]. The gene-specific oligonucleotides used in the present study are listed in Supplementary Table S1.

### 4.7. Root Hair Measurements

The number of root hairs was determined in 1 mm long sections within the root hair elongation zone and root hair mature zone of the control, *CRK12*-OE and *CRK12*-RNAi transgenic hairy roots at 10 days post emergence. The root hair length was measured using the LAS EZ 3.4.0.272 suite, and the density was calculated according to the methods of Ma et al. [60].

### 4.8. Nodule Phenotype and Microscopy

The uninoculated and *R. tropici* CIAT899 (wild type)-inoculated transgenic root samples in which p*CRK12*::GUS-GFP or *CRK12* was overexpressed or *CRK12* silenced were inoculated with *R. tropici* CIAT899 expressing GUS (harbouring pGUS 32) [61] or CIAT899 expressing RFP. The GUS assay was performed as described by Jefferson [62]. Root and nodule tissues were transferred to a 3 cm diameter Petri dishes containing a GUS staining solution and incubated at 37 °C in the dark until blue or magenta spots were visible (approximately 8–12 h). The GUS-stained roots were clarified using 0.5% sodium hypochlorite for 8 h and then examined for nodule symbiosis phenotype, viz., infection threads, nodule primordia, and nodules, under a light microscope (Leica, DMLB bright-field microscope, Buffalo Grove, IL, USA). The GUS-stained mature nodules were sectioned using a razor blade. The sections were mounted in 10% glycerol and observed under a light microscope. The GUS-stained transgenic roots expressing the p*CRK12*::GUS-GFP promoter were observed under a stereomicroscope (Leica), and images of young and mature nodules were obtained.

The transgenic roots that expressed subcellular localization construct or the *CRK12* promoter construct was observed under a Zeiss LSM 710-NLO confocal microscope (Centro de Investigaciones en Optica A.C., León, Mexico) with a 25X 0.8 NA oil immersion objective. GFP was excited with an argon laser (488 nm), and the fluorescence from 510 to 540 nm was recorded. YFP was excited with the 514 nm line of the argon laser, and the fluorescence from 519 to 620 nm was recorded. RFP was excited with a solid-state laser (561 nm), and the fluorescence was filtered using a 640-/650-nm bandpass filter. The nodule cross sections were obtained by paraffin embedding and semithin sectioning following the protocol by Chen et al. [63]. The tissues were fixed in FAA fixative containing 10% formalin, 5% glacial acetic acid, 50% ethanol and 35% deionized water. Post fixation, the tissues were processed through a dehydration series of ethanol solutions (from 30% to 100%). The paraffin embedded tissues were sectioned using the Leica RM2125 RTS microtome and stained with 0.1% Toluidine blue. Acetylene reduction assay was performed following the method described by Burris [64]. 21 dpi nodulated transgenic roots of control, *CRK12*-RNAi and CRK12-OE were incubated in acetylene gas for 30, 60 and 90 min, and the ethylene production was measured by gas chromatography in a Hewlett Packard 5890 Series II (Wilmington, DE, USA).

### 4.9. Statistical Analysis

All the statistical data were analyzed by Student’s *t* test using Prism 9.0 software (GraphPad Software Inc., Boston, MA, USA). In the figures, single, double, and triple asterisks were used indicate differences that are significant (*p* < 0.05 or *p* < 0.01) or highly significant (*p* < 0.001), respectively.

## Data Availability

Data are contained within the article and Supplementary Materials.

## Supplementary Materials

The following supporting information can be downloaded at: https://www.mdpi.com/article/10.3390/ijms241411720/s1.
