# Supplementary material for: CRK12: A Key Player in Regulating the Phaseolus vulgaris-Rhizobium tropici Symbiotic Interaction
Source: Int J Mol Sci. 2023 Jul 21;24(14):11720. doi: 10.3390/ijms241411720 (PMC10380779; doi:10.3390/ijms241411720)
Supplement: Supplementary file 1 [file ijms-24-11720-s001.zip › Supplementary Figure S1.pdf]

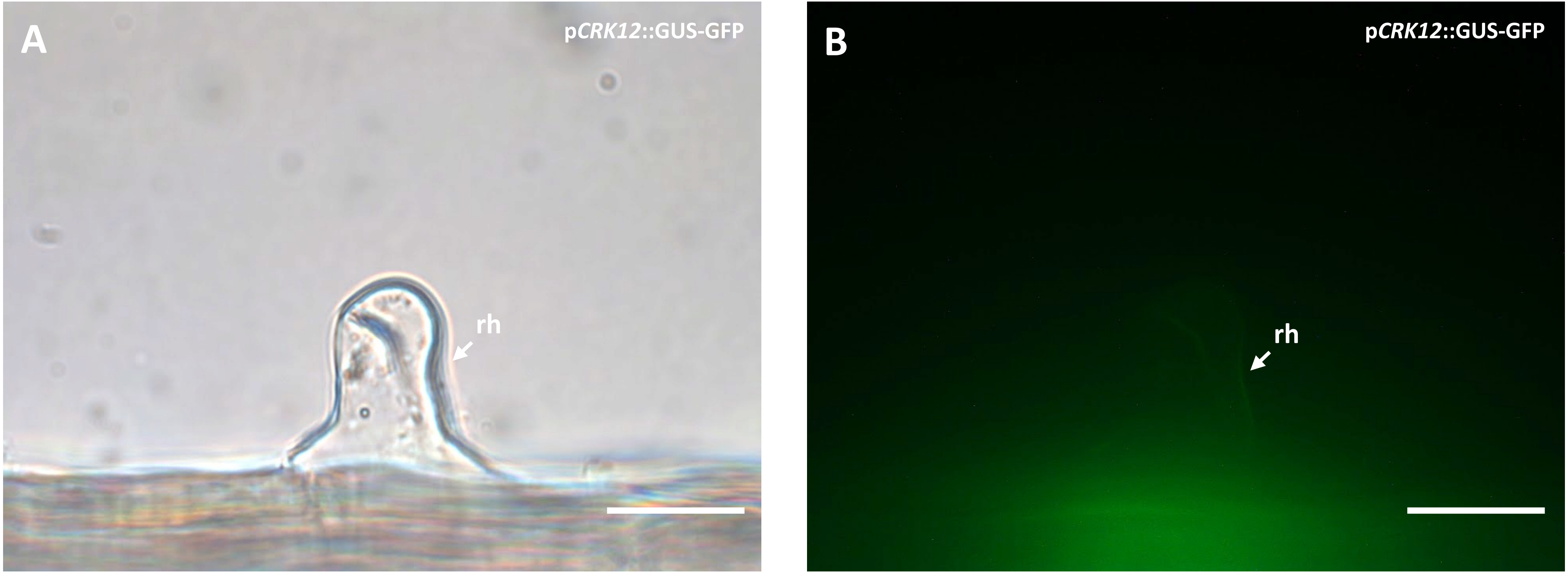

Supplementary Figure S1: Spatiotemporal expression patterns of *CRK12* promoter in root hair cell of uninoculated *Phaseolus vulgaris* roots. The transgenic hairy roots expressing *pCRK12*::GUS-GFP construct and observations were recorded using fluorescent microscope at 3 dpi. The representative root hair cell **A)** in white light, and **B)** pCRK12 activity detected as GFP fluorescence. rh, root hair. Bars 20 µm.
