# Supplementary material for: CRK12: A Key Player in Regulating the Phaseolus vulgaris-Rhizobium tropici Symbiotic Interaction
Source: Int J Mol Sci. 2023 Jul 21;24(14):11720. doi: 10.3390/ijms241411720 (PMC10380779; doi:10.3390/ijms241411720)
Supplement: Supplementary file 1 [file ijms-24-11720-s001.zip › Supplementary Table S1 .pdf]

Supplementary Table S1: Primer sequences of *Phaseolus vulgaris* genes used to generate constructs and perform quantitative RT-PCR.

Phaseolus vulgaris **gene**	**Oligonucleotide sequence (5' – 3')**	
Promoter *CRK12*	**F**	GACCAATGTGAGACAGGCC
	**R**	GCAGAAAGAATATCCCAAAAC
CDs *CRK12*	**F**	CATGGTTTCTGTGATCCGTCTG
	**R**	GTTGGTCTGAACGATACGAG
CRK12-RNAi	**F**	GGAGAAGGTGGTTTTGGAGC
	**R**	CTCGTATCGTTCAGACCAAC
qPC*R CRK12*	**F**	GGAGAAGGTGGTTTTGGAGC
	**R**	GTTGGTCTGAACGATACGAG
EIF4a	**F**	GGTGTCCACACTGTTGTTGG
	**R**	CGGCATTGTAGCAGAGAACA
IDE	**F**	GCAACCAACCTTTCATCAGC
	**R**	AGAAATGCCTCAACCCTTTG
RGF6	**F**	GCTACAGGAAAATCAACAGCAGCAAG
	**R**	CTGACCAGCTCCCAGCATTGACC
RGF9	**F**	AGGCCATGTCTTGTGCAAGTGCC
	**R**	TTGTTGTGCAGCTTCATGTTGTGGC
RbohB	**F**	GGAAGGAGATGCTCGATCTGC
	**R**	GTCTTCACCCTTGTCCCTGAAAC
BPS1.1	**F**	AGCTTTCACAGGGACTAGATC
	**R**	CAAACTTCAATGAGCACAGGTAAC
ARF5	**F**	GGCCGGTTCTGTTGGAAG
	**R**	CACCCTTGACTCATTGGAAC
ARF7	**F**	GGGGTTTTGAATAATGGCTTGTGGGC
	**R**	GCTCCAAAATGTCAATACTAGACATCC
YUCCA	**F**	GCAGTGGTGCTTGCAACCGG
	**R**	CTATGACAAGCAGTAGTGCGC
RSL2	**F**	CGGGTGAATTTCGCAACGGAG
	**R**	GGACAAGCATGACCACACACC
CPC (CAPRICE)	**F**	ATGTCCACCACCGCAACTTC
	**R**	CCATGAGAGATTGTTGAGGAG
SymRK	**F**	GAAGATTTATGGTACTAGGT

	**R**	TGTCAAGGCTACTCTGGA
CCamK	**F**	GTGCATCACCAAGGAAGAAGTAG
	**R**	TCACTGTTGGCATCCATTCTGTC
NSP2	**F**	ACTATTCGGCGGTGTTTGAC
	**R**	TAGAAGCTTCGCTTGGCAAT
NIN	**F**	GGGGATTCAGAGATTTGCAG
	**R**	AACCCACTCTTGAGCATCGT
Enod40	**F**	AGTTTTGTTGGCAAGCATCC
	**R**	TAAGCACAAGCAAACTGTTG
RACK1	**F**	GGCTCTATTATCCATGCCC
	**R**	GGCCTCAAAGCCACGGTC
GOGAT	**F**	ATGTCTTCATCAATCTCGTTTCCCACG
	**R**	ACGCAACCGTGTTCCCAAAAACTTTGTC
PRAT3	**F**	GGTTGAAGATGCTGTTGGAG
	**R**	CCACTTAGCTTACCCAACG
